# Oxidized Fatty Acids as Inter-Kingdom Signaling Molecules

**DOI:** 10.3390/molecules19011273

**Published:** 2014-01-20

**Authors:** Carolina H. Pohl, Johan L.F. Kock

**Affiliations:** Department of Microbial, Biochemical and Food Biotechnology, University of the Free State, PO Box 339, Bloemfontein 9300, South Africa; E-Mail: KockJL@ufs.ac.za

**Keywords:** oxidized fatty acids, signaling, inter-kingdom communication

## Abstract

Oxylipins or oxidized fatty acids are a group of molecules found to play a role in signaling in many different cell types. These fatty acid derivatives have ancient evolutionary origins as signaling molecules and are ideal candidates for inter-kingdom communication. This review discusses examples of the ability of organisms from different kingdoms to “listen” and respond to oxylipin signals during interactions. The interactions that will be looked at are signaling between animals and plants; between animals and fungi; between animals and bacteria and between plants and fungi. This will aid in understanding these interactions, which often have implications in ecology, agriculture as well as human and animal health.

## 1. Introduction

Until recently the study of signaling and response in different kingdoms of life developed independently from each other, with little regard for the similarities between the different kingdoms. However, it has become apparent that elements of signaling are shared by different kingdoms [1,2,3,4]. It is speculated that many of these are ancestral traits or that some may be due to convergent evolution, horizontal gene transfer or ancient symbiosis [1]. These shared signals come into play when there is interaction between members of the different kingdoms (ranging from mutually beneficial interactions such as symbiosis, to ones harmful to at least one member such as parasitism), and include plant defenses against herbivores and plant pathogens, animal defenses against pathogenic microbes and parasites as well as the influence of bacterial and fungal quorum sensing molecules on members of the different kingdoms. Schultz and Appel [2] speculated that the ability of interacting organisms to communicate through shared signal systems may provide an adaptive advantage and that there are a limited number of these signaling systems. When one starts to examine the shared signals involved in these interactions, it becomes evident that a group of signals based on fatty acids are often shared between different kingdoms [3]. Enzymatically modified lipids have ancient evolutionary origins as signaling molecules [5] and are ideal candidates for communication with and manipulation of interacting parties.

The aim of this review is to discuss examples of the ability of organisms from different kingdoms to “listen” and respond to fatty acid signals, specifically oxidized fatty acid, during interactions. This will aid in understanding these interactions, which often have implications in ecology, agriculture as well as human and animal health.

## 2. Inter-Kingdom Signaling between Animals and Plants

Most of the study of inter-kingdom signaling between animals and plants focuses on the plant’s response to herbivores, such as insects. These responses are often due to hormones that are shared between the different kingdoms [2]. In addition, it is well known that plants and animals produce a range of similar oxidized fatty acids (oxylipins/eicosanoids). These oxidized fatty acids are considered central to inter-kingdom interactions involving plants.

**Figure 1:**
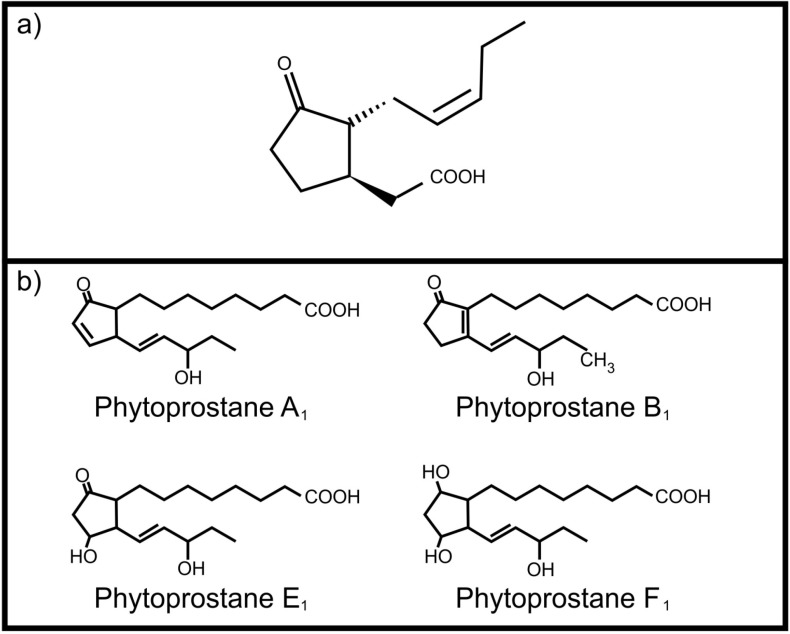
Plant oxylipins involved in communication with animals (**a**) Jasmonic acid; (**b**) Phytoprostanes.

One of the most important plant oxylipins is jasmonic acid (Figure 1a). It regulates several important physiological processes in plants including induced defense against herbivores [6]. It is produced from α-linolenic acid upon wounding and leads to the production of toxic compounds as protective measure. Although it mainly serves as an internal signaling molecule, Li and co-workers [7] reported that jasmonic acid, ingested by the corn ear worm (*Helicoverpa zea*), activates transcription of four cytochrome P450 genes involved in metabolism of plant toxins. This ability to “listen” to plant oxylipin signals protects *H. zea* against the host plant’s defenses. Plants also have the ability to produce autoxidation products, the phytoprostanes, from α-linolenic acid. These include phytoprostane E_1_ [8], phytoprostane F_1_ [9] phytoprostane A_1_ and phytoprostane B_1_ [10] (Figure 1b). Of specific interest is the occurrence of these phytoprostanes in pollen, where they are known collectively as pollen-associated lipid mediators (PALMs) [11]. When pollen comes into contact with mucous membranes of animals these PALMs are released. Certain PALMs are immunostimulatory in humans, activating polymorphonuclear granulocytes, neutrophils and eosinophils [11,12]. In addition, phytoprostane E_1_ modulates cytokine (IL-12) production by dendritic cells through a PPAR-γ dependent pathway that leads to inhibition of NF-κB activation. This results in an increased Th 2 response, such as inflammation, mediated by the release of Th 2 cytokines (e.g., IL-4), as well as activation of eosinophils, mediated by IL-5, which is characteristic of pollen allergy [13].

**Figure 2:**
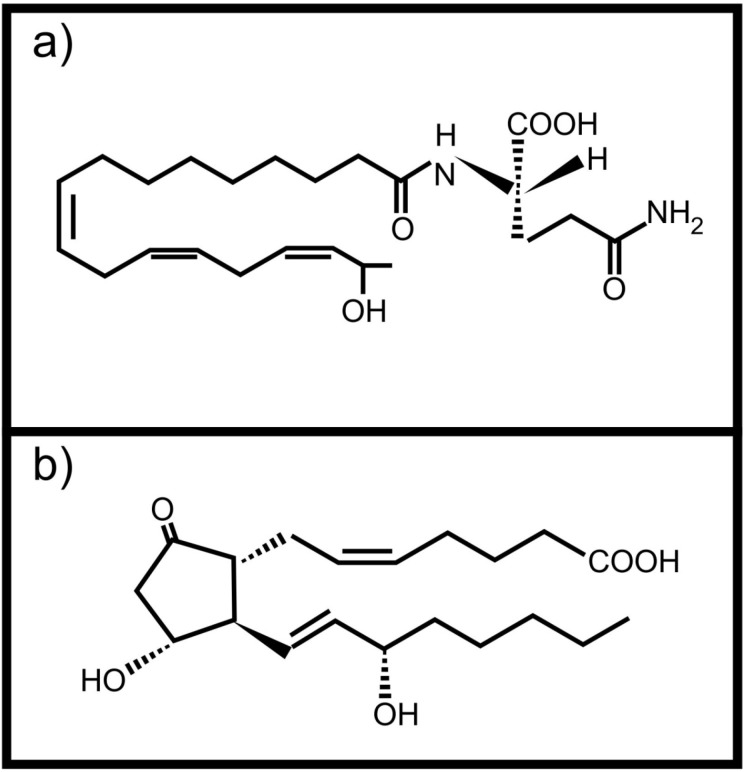
Animal derived oxidized fatty acid signals involved in communication with plants (**a**) Volicitin; (**b**) Prostaglandin E_2_.

Plants can also perceive fatty acid signals from animals. Volicitin (Figure 2a) is a conjugate of 17-hydroxy linolenic acid and L-glutamine produced and secreted by caterpillars [14]. It comes into contact with the plant when the caterpillars feed and results in the production of plant protective volatile compounds in some plants [14,15,16]. It was also shown that plants wounded and treated with caterpillar regurgitant, containing volicitin, increased their foliar tannin concentration without a negative effect on growth [17]. This is not the only oxidized fatty acid based molecule with potential inter-kingdom signaling activity found in insect regurgitant. Schultz and Appel [2] showed that tannin production by plants after wounding, is suppressed when prostaglandin E_2_ (Figure 2b, an animal derived arachidonic acid metabolite) is added to the wound. They also showed that the same effect was observed when the wounded plants were treated with regurgitant of the gypsy moth or forest tent caterpillars, speculated to contain prostaglandin E_2_. Unfortunately the exact composition of the caterpillar regurgitant was not determined.

This inter-kingdom signaling between plants and animals is also found between diatoms (unicellular algae) and small crustaceans, the copepods, that feed on them. Several diatoms (e.g., *Pseudo-nitzchia delicatissima, Chaetoceros* spp., *Thalassiosira rotula, Cerataulina pelagica*) produce a range of oxylipins, including hydroxy fatty acids. Many of these oxylipins have a negative impact on copepod egg production, hatching success, and development of the offspring, similar to the more common diatom toxins, the polyunsaturated aldehydes, although the precise mechanism is still unknown [18,19,20]. It is speculated that oxylipins as signaling molecules are so fundamental to survival of plants, where they play a crucial role in defense, that they have been conserved through evolution [20].

## 3. Inter-Kingdom Signaling between Animals and Fungi

Animals produce an range of oxygenated C_20_ fatty acids, the eicosanoids, which include prostaglandins, thromboxanes, prostacyclins, leukotrienes, lipoxins, hepoxilins, hydro(pero)xy fatty acids, hydroxylated and epoxy fatty acids [21,22]. They are produced by cyclooxygenases [23], lipoxygenases [24], cytochrome P450s [22,25], or nonenzymatic pathways [26] from fatty acid precursors, dihomo-γ-linolenic acid, arachidonic acid, eicosapentaenoic acid [21] and docosahexaenoic acid [27]. The immunomodulatory properties of eicosanoids have been studied intensively in mammalian cells with a single eicosanoid capable of having pleiotropic functions [28,29]. These effects are mainly due to the existence of multiple G-protein-coupled receptors (GPCRs), known as guanine nucleotide regulatory proteins, for each lipid species [21,29]. The activated trimeric G-proteins affect the concentrations of the second messengers, cyclic AMP (cAMP), or intracellular ions such as K^+^.

**Figure 3:**
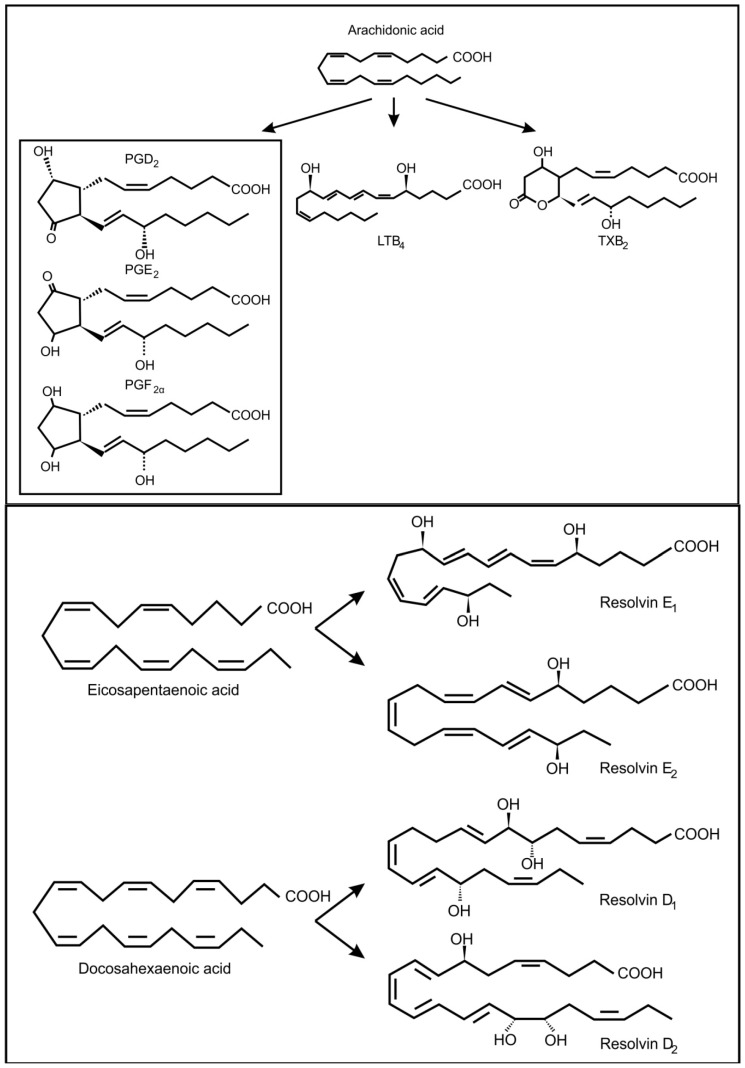
Oxidized fatty acids derived from arachidonic acid as well as from eicosapentaenoic acid and docosahexaenoic acid, involved in inter-kingdom signaling between fungi and animals.

In fungi, the precursors for oxylipin production are usually oleic acid, linoleic acid and α-linolenic acid [30]. However, it is known that pathogenic yeasts can produce oxylipins from arachidonic acid, which they may acquire from the infected host cell (Figure 3) [31]. In the genus *Candida*, several potentially pathogenic species (*i.e*., *C. albicans, C. dubliniensis*, *C. glabrata* and *C. tropicalis*) can produce prostaglandin E_2_ (PGE_2_) [32,33,34]. *Candida albicans* can also produce prostaglandin D_2_ (PGD_2_), prostaglandin PGF_2α_ (PGF_2α_) and leukotrienes (LTB4, cysteinyl leukotrienes) from arachidonic acid [35]. *Cryptococcus neoformans*, is also capable of producing PGE_2_, PGD_2_, PGF_2α_ and leukotrienes [32,35] and *Paracoccidioides brasiliensis* can use exogenous or endogenous arachidonic acid to produce prostaglandin E_x_ (possibly PGE_2_) [36,37]. Another important fungal respiratory pathogen, *Aspergillus fumigatus* and *Aspergillus nidulans* contain cyclooxygenase like enzymes and are also capable of producing arachidonic acid metabolites [38,39]. These included PGE_2_, 6-keto-prostaglandin F_1α_, PGF_2α_, isoprostanes and thromboxane B_2_, most of which decrease the pulmonary function of the host. *Candida albicans* can also produce eicosanoids from eicosapentaenoic acid and docosahexanoic acid (Figure 3) [5]. One of these eicosanoids, resolvin E_1,_ is a potent anti-inflammatory lipid that attenuates neutrophil migration during the resolution phase of inflammation. Is has been suggested that low levels of resolvin E_1_, produced by commensal *C. albicans,* would dampen the adaptive immune response and protect the commensal yeast from the host’s immune response.

Since both host and pathogen are capable of producing eicosanoids during an infection these signals may be involved in a complex inter-kingdom dialogue between animal (host) and fungus. The enhanced production of prostaglandins and leukotrienes by pathogenic yeasts and the biological effects of prostaglandins on the host immune system may lead to the intracellular survival followed by chronic and disseminated infections [36,38,40,41,42,43]. It is also tempting to speculate that fungal spores contain lipid mediators that are released upon contact with mucous membranes and may elicit responses associated with disease and/or allergies in a similar manner to PALMs in plant pollen.

In addition, host derived eicosanoids have an effect on yeast cells. Prostaglandin E_2_ induces morphogenesis (*i.e*., yeast-to-hyphae transition) in *C. albicans* and *C. dubliniensis*, probably by increasing cellular cAMP levels [34,44,45]. Similarly thromboxane B_2 _(TXB_2_) also increases morphogenesis in *C. albicans* [45].

## 4. Inter-Kingdom Signaling between Animals and Bacteria

**Figure 4:**
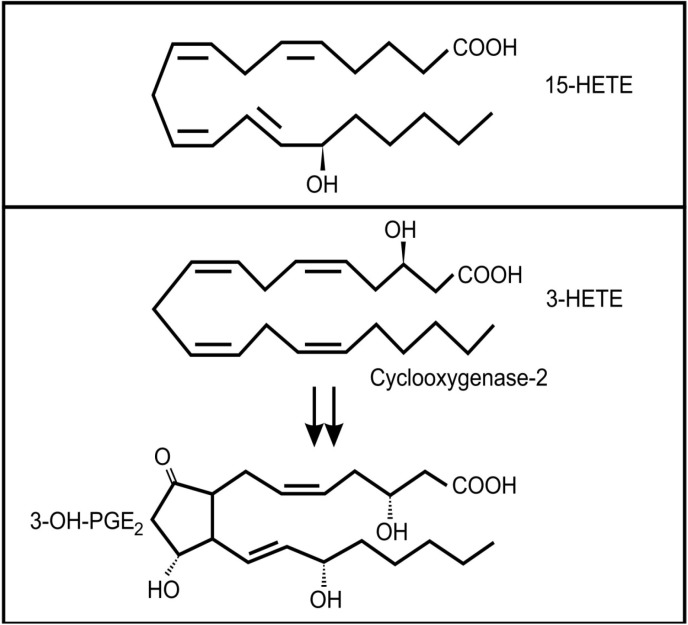
Oxidized fatty acids involved in cross-kingdom signaling between bacteria and animals (**a**) 15-hydroxyeicosatetraenoic acid (13-HETE); (**b**) The conversion of 3-HETE to 3-OH-prostaglandin E_2_.

The lung secretions of cystic fibrosis (CF) sufferers contain a wide range of inflammatory and anti-inflammatory oxidized fatty acids, including arachidonic acid metabolites [46]. It is known that several bacterial species can infect CF lungs and that these microbes may contribute to the production of these lipid mediators [47]. *Pseudomonas aeruginosa* is the most important bacterial colonizer of CF lungs [48]. Interestingly, it was found that a virulence factor in *P. aeruginosa*, the type III secretion effector molecule, ExoU, induced the release of arachidonic acid from human endothelial cells [49]. In addition, this bacterium has the ability to convert arachidonic acid to 15-hydroxyeicosatetraenoic acid (15-HETE) (Figure 4a) through a secreted 15-lipoxygenase [50]. 15-HETE can activate several mammalian signaling pathways, including MAP kinases and at high concentrations it can activate the PPAR-γ pathway, which plays an important anti-inflammatory role via inhibition of NF-κB expression [51]. Although the role of 15-HETE in airway epithelium is not clear, there has been suggestions that it could play a role in mucous production and bronchial contractibility [52].

Another bacterium often associated with the lungs of adult CF patients is *Stenotrophomonas maltophilia* [48]. Weil and co-workers [53] showed that this bacterium can produce 3(*R*)-hydroxy fatty acids from a range of precursors by a process analogous to β-oxidation. Although they did not test arachidonic acid as a precursor, if their conclusions are applied to the oxidation of arachidonic acid, the product would be 3(*R*)-hydroxyeicosatetraenoic acid (3-HETE). Mammalian cyclooxygenase 2 (COX-2) can oxygenate 3-HETE to 3-OH-prostaglandin E_2_ (Figure 4b) which is a more potent inducer of proinflammtory interleukin 6 (IL-6) mRNA expression than PGE_2_ [54]. It is interesting to note that IL-6 is one of the important inflammatory markers in patients with CF, especially during episodes of acute pulmonary exacerbations [55,56] and levels of this cytokine is correlated with loss of fat-free mass in these patients [57]. Whether the increased level of IL-6 in these patients is influenced by *Stenotrophomonas maltophilia* infection still needs to be determined.

## 5. Inter-Kingdom Signaling between Plants and Fungi

**Figure 5:**
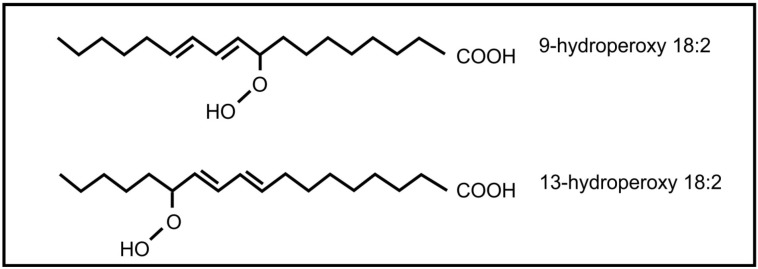
Plant oxylipins involved in communication with fungi.

The roles of oxylipins in plant-fungal interactions were reviewed by Christensen and Kolomiets [58]. Plant oxylipins can influence reproduction in fungi. Examples include the plant lipoxygenase products 9-hydroperoxy octadecadienoic acid and 13- hydroperoxy octadecadienoic acid (Figure 5) that can induce conidial development in several *Aspergillus* species. At low concentration 9-hydroperoxyoctadecadienoic results in sexual spore formation instead of conidial development in *Aspergillus nidulans* [59]. These oxylipins also influence mycotoxin production by *Aspergillus*, with 9-hydroperoxyoctadecadienoic acid stimulating toxin production and 13-hydroperoxyoctadecadienoic acid inhibiting toxin production. Evidence suggests that this regulation is transcriptional [60]. It is further speculated that 9-lipoxygenase derived plant oxylipins act as fungal signals that regulate pathogenicity, spore and toxin production by several plant pathogenic *Aspergillus* species and *Fusarium verticilloides* [61]. The jasmonic acid metabolite, methyl jasmonate, was also found to regulate reproduction and toxin production in fungi. It decreases sporulation and aflatoxin production in *Aspergillus flavus* [62] and stimulates aflatoxin production in *Aspergillus parasiticus* [63].

**Figure 6:**
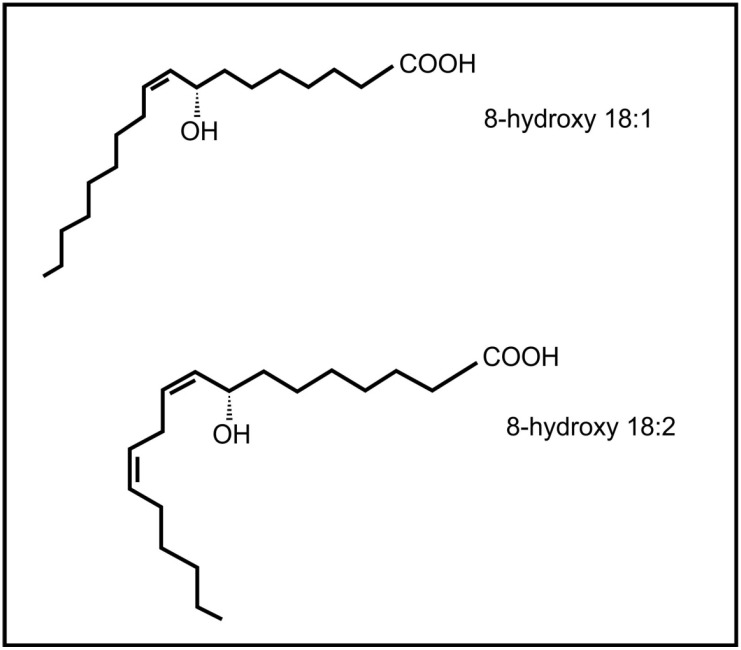
Fungal oxylipins involved in communication with plants.

Brodhagen and co-workers [64] also demonstrated that the communication between plants and fungi is not one way, but that fungal oxylipins (e.g., 8-hydroxyoctadecenoic acid and 8-hydroxy-octadecadienoic acid) (Figure 6) also influence expression of plant genes, *i.e*., plant lipoxygenase genes. The plant pathogenic fungus, *Lasiodiplodia theobromae* is able to produce the plant oxylipin jasmonic acid [65]. The release of fungal jasmonic acid during infection leads to the inhibition of the salicylic acid mediated defense system in the plant, contributing to infection by this fungus.

**Figure 7:**
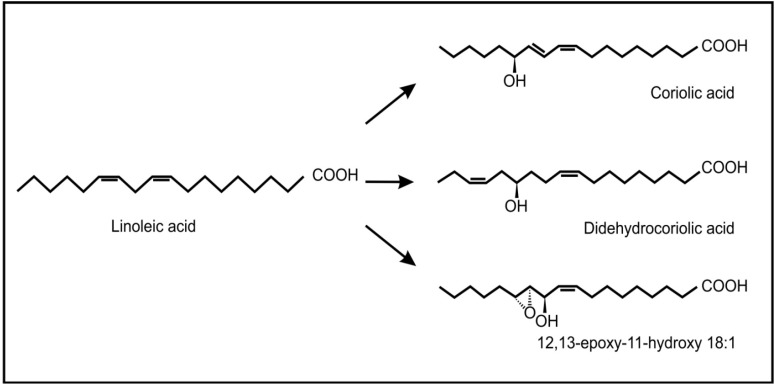
Typical plant oxylipins produced by the fungus, *Fusarium incarnatum,* from linoleic acid.

Recent evidence has also shown the occurrence and possible role of oxylipins during beneficial plant-fungus interactions. Products of the 9-lipoxygenase pathway that normally serve as plant defense compounds, were also found to be involved in regulation of symbiotic fungal growth during arbuscular mycorrhiza development in tomatoes [66]. Another interesting example is the endophytic fungus, *Fusarium incarnatum*, found inside the embryos of the mangrove tree *Aegiceras corniculatum* [67]. This fungus can produce archetypal plant defense oxylipins (coriolic acid, didehydrocoriolic acid and 12,13-epoxy-11-hydroxyoctadecenoic acid) from linoleic acid by the action of a desaturase and a 13-lipoxygenase (Figure 7). These authors speculated that these fungal oxylipins may serve to protect the embryos, during dispersal by sea and so improve the chances of reproduction.

## 6. Conclusions

Oxidized fatty acids are present in diverse kingdoms of life, and organisms from these different kingdoms have the ability to produce, detect and respond to these inter-kingdom signaling molecules. These responses are varied and may be involved in pathogenesis or in benign, symbiotic interactions. As such they play important roles in ecology, agriculture and medicine. Although there are still many unanswered questions regarding the specific signals and their mechanism of action, understanding this and other inter-kingdom signals will increase our understanding of these interactions.
